# Unique Design of Functionalized Covalent Organic Frameworks for Highly Selective Removal of Cyano-Neonicotinoids

**DOI:** 10.3390/nano15201596

**Published:** 2025-10-20

**Authors:** Yan Yang, Shuojie Wang, Wenxin Mai, Shiyu Wei, Guixiang Teng, Peng Pu, Jiaxing Zhao, Yongqiang Tian

**Affiliations:** School of Biological and Pharmaceutical Engineering, Lanzhou Jiaotong University, Lanzhou 730070, Chinawsy@lzjtu.edu.cn (S.W.); tenggx@lzjtu.edu.cn (G.T.); pupeng@lzjtu.edu.cn (P.P.); zhaojiaxing@lzjtu.edu.cn (J.Z.)

**Keywords:** covalent organic frameworks, neonicotinoid, selective removal, Au nanoparticles, adsorption mechanism, food safety

## Abstract

Acetamiprid (ACE) and thiacloprid (THIA) are the dominant cyano-substituted neonicotinoids detected in fruit juices and bottled water, which raises food-safety concerns and regulatory scrutiny. Conventional purification with activated carbon or advanced oxidation shows limited selectivity and has a high energy demand. Covalent organic frameworks (COFs) offer tunable chemistry for targeted adsorption, yet no strategy exists to engineer COF sites that preferentially recognize the cyano group of ACE/THIA. Here, we synthesized a magnetic core-shell adsorbent, Fe_3_O_4_@COF(TBTD-BD)-Au, by growing cyano-affinitive Au nanoparticles on a Cl-decorated COF shell surrounding a Fe_3_O_4_ core. Under optimized conditions (pH 6.0, 25 °C), the Fe_3_O_4_@COF(TBTD-BD)-Au achieved maximum adsorption capacities of 157 mg g^−1^ (ACE) and 156 mg g^−1^ (THIA). Uptake followed pseudo-second-order kinetics and the Freundlich isotherm; thermodynamic analysis confirmed an endothermic, spontaneous process. Competitive tests showed >80% removal of ACE and THIA in the presence of four co-occurring neonicotinoids, and the adsorbent retained 91.5% of its initial capacity after six adsorption–desorption cycles. Synergistic Au-cyano coordination, Cl-mediated hydrogen bonding, and π–π stacking confinement confer high selectivity and capacity. This ligand-guided, post-functionalized COF provides promising potential in the field of food sample treatment for contaminant removal.

## 1. Introduction

Neonicotinoid insecticides are one of the most widely utilized pesticides globally for controlling sap-sucking insects by interfering with their nervous systems [1]. Acetamiprid (ACE) and thiacloprid (THIA) are extensively used in agricultural pest management, yet mounting evidence links their overuse to adverse human-health and environmental outcomes [2]. Notably, their propensity for bioaccumulation and high toxicity pose grave risks to ecosystems, threatening various components of the food chain [3,4]. Consequently, devising a selective and effective method for removing these neurotoxic compounds from complex matrices is critically important.

Several methods have been explored for the removal of ACE and THIA from matrices, such as microbial and oxidative degradation [5,6], electrocoagulation, photolysis [7], and adsorption [8]. Electrocoagulation, electro- or photolysis, and degradation suffer from high energy demand, complex pretreatment steps, and the generation of several toxic and persistent coproducts, which restricts their widespread application. By contrast, adsorption offers operational simplicity and efficiency [9] while avoiding secondary pollution from degradation by-products [10]. However, conventional adsorbents typically face challenges like inadequate selectivity, limited regeneration capacity, and low adsorption efficiency [11], which creates an urgent demand for the development of a new adsorbents with powerful adsorption performance, remarkable repeatability, and high selectivity for the removal of neonicotinoid insecticides from the food matrix. Among tailored materials, Au/Ag-based adsorbents often rely on non-specific surface interactions and are susceptible to matrix interference. MIP systems provide tailored binding sites, but they frequently suffer from template leakage and reduced selectivity when used in complex sample matrices. Likewise, high-surface-area MOFs without further functionalization do not inherently target the cyano group [12]. COFs, characterized by their periodic, porous structure, extensive surface area, and numerous functional groups along their channel walls, are exceptional candidates for roles in separation, adsorption, catalysis, and sensing [13,14,15]. Despite the large number of published works on synthetic reactions and applications, constructing functional COFs that can achieve highly selective and efficient removal for targeted analytes remains a challenge [16,17]. Existing efforts emphasize post-synthetic modification, whereas systematic ligand-level design is comparatively under explored. Guided by analyte structure, integrating ligand engineering with post-synthetic functionalization to construct multi-functional COFs that prearrange recognition elements offers a promising route to structurally precise platforms capable of high-affinity enrichment in complex matrices.

ACE and THIA feature electron-deficient chloro(hetero)aryl rings, a nitrile (–C≡N) handle, and hydrophobic substituents, which suggests that COFs with programmable pore chemistries are well suited for their capture. The cyano group offers a soft-base site that can coordinate to soft Lewis-acidic Au, while chlorine substituents can engage in weak C–H···Cl hydrogen-bonding/halogen-bond-like contacts; together with π–π and hydrophobic interactions, these motifs inform a rational adsorption blueprint [18]. We therefore embedded Au nanoparticle sites within chlorine-decorated COF channels by using a TBTD-BD ligand set, which yielded a multifunctional adsorbent in which co-localized Au sites and Cl-rich microenvironments cooperatively enhanced the selective removal of ACE and THIA from complex matrices.

**Figure 1:**
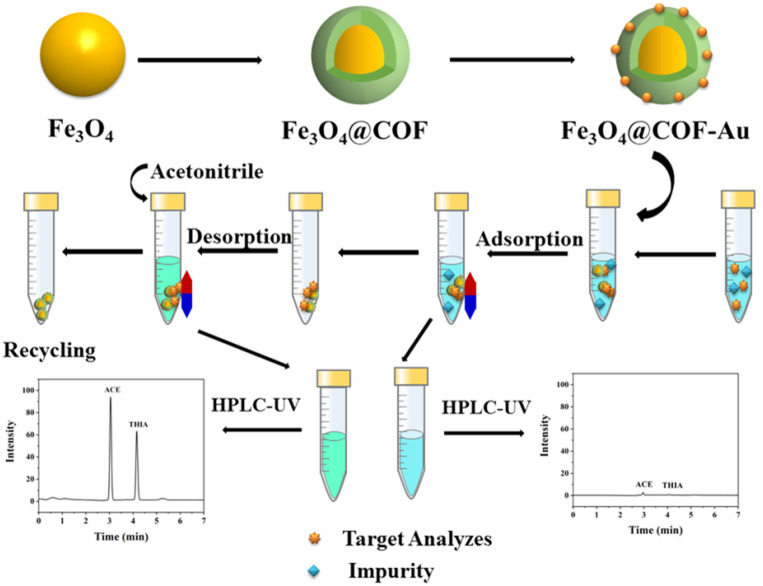
Schematic fabrication process of Fe_3_O_4_@COF(TBTD-BD)-Au and its application to analyte removal.

Here, we designed and synthesized magnetic Cl-decorated porous COFs by incorporating Fe_3_O_4_ as magnetic cores and using 2,4,6-trichloro-benzene-1,3,5-tricarbaldehyde (TBTD) and benzidine (BD), designated as Fe_3_O_4_@COF(TBTD-BD), as two building units. Au nanoparticles were integrated into Fe_3_O_4_@COF(TBTD-BD) networks by self-assembly. Unlike conventional carbon adsorbents that exhibit nonspecific uptake in complex matrices, this material is pre-organized with Cl-decorated channels and subsequently endowed with site-addressable Au, which enables cooperative, multimodal recognition of ACE/THIA through its Au–nitrile affinity together with its C–H···Cl hydrogen-bond/halogen-bond-like contacts, π–π stacking, and hydrophobic interactions. The magnetic Fe_3_O_4_ core enables facile separation under an external magnetic field, while the functional groups in the porous COF (TBTD-BD)-Au furnish abundant binding sites, thereby ensuring selective and efficient adsorption of ACE and THIA. Figure 1 illustrates the fabrication of Fe_3_O_4_@COF(TBTD-BD)-Au and the procedure for analyte removal.

To evaluate the potential of Fe_3_O_4_@COF(TBTD-BD)-Au in removing ACE and THIA, parameters (such as pH, ionic strength, and contact time) influencing the removal capacity were optimized. Moreover, kinetic, isotherm, thermodynamic, and reutilization studies were also performed to characterize the use of Fe_3_O_4_@COF(TBTD-BD)-Au during the ACE and THIA removal process. Furthermore, the main interactions between analytes and adsorbents were also evaluated. The large adsorption capacity, high selectivity, rapid adsorption kinetics, excellent stability, and good reusability of Fe_3_O_4_@COF(TBTD-BD)-Au demonstrate its significant potential for targeted removal from complex matrices.

## 2. Materials and Methods

### 2.1. Chemicals

All reagents were used as received without further purification. The standards of ACE (99.5%), THIA (98%), thiamethoxam (THI, 98%), imidacloprid (IMI, 98.7%), clothianidin (CLO, 99.3%), nitenpyram (NIT, 99.1%), and 2,4,6-trichloro-benzene-1,3,5-tricarbaldehyde (TBTD, >98%) were purchased from Aladdin (Aladdin Industrial Corporation, Shanghai, China). Benzidine (BD), 1,3,5-triformylphloroglucinol (TP), sodium borohydride (NaBH_4_), and chloroauric acid (HAuCl_4_·4H_2_O) were obtained from Sigma-Aldrich (Shanghai, China). Ferric trichloride hexahydrate (FeCl_3_·6H_2_O), ammonium acetate (NH_4_Ac), ethylene glycol, sodium citrate, and ammonium hydroxide (NH_3_·H_2_O) were purchased from Sinopharm Chemical Reagent Co., Ltd. (Shanghai, China). All other materials were of analytical reagent grade and obtained from the Beijing Chemical Reagent Factory (Beijing, China).

### 2.2. Preparation of Fe_3_O_4_, Fe_3_O_4_@COF, and Fe_3_O_4_@COF-Au

The synthesis of Fe_3_O_4_ was carried out according to a previously reported method [19], with minor modification. FeCl_3_·6H_2_O (1.35 g), NH_4_OAc (3.85 g), and sodium citrate (0.4 g) were dissolved in ethylene glycol (80 mL) under vigorous magnetic stirring (1 h) to form a black solution. Then, the solution was transferred to a 100 mL Teflon-lined stainless-steel autoclave. After reaction at 200 °C for 16 h, the black precipitate was magnetically separated, washed several times with water and ethanol until the supernatant was clear, and subsequently dried under vacuum at 60 °C for further use.

The obtained Fe_3_O_4_ nanoparticles were further coated with COF shells by the Schiff-base reaction of TBTD and BD. Typically, TBTD (52 mg) and Fe_3_O_4_ (80 mg) were dispersed in 20 mL of DMSO by ultrasonication for 10 min to obtain a homogeneous solution. Subsequently, BD (55 mg) and acetic acid (1.8 mL) were added dropwise under ultrasonication, after which the mixture was sealed and kept at room temperature for 2 h without stirring. Finally, the claybank substances (Fe_3_O_4_@COF) were collected by a magnet. The product was sequentially washed several times with THF and MeOH until the supernatant became clear. After that, the precipitates were dried under vacuum at 60 °C for further use (yield, 90%). The Fe_3_O_4_@COF(TPBD) was synthesized according to methods that have been previously reported [20]. Detailed synthetic procedures are provided in the Supplementary Information.

The obtained Fe_3_O_4_@COF(TBTD-BD) nanoparticles were functionalized with Au nanoparticles. A total of 50 mg Fe_3_O_4_@COF(TBTD-BD) was dispersed in 15 mL of ethanol solution containing 0.02% HAuCl_4_ and 0.02% tri-sodium citrate, and the reaction was allowed to proceed under mechanical stirring for 2 h. Next, 2 mL of fresh 0.38% NaBH_4_ solution was dropwise added to the solution and further stirred for 3 h. Finally, the products were washed several times sequentially with ethanol and ultrapure water, and then dried at 80 °C under vacuum overnight (yield, 95%). The Fe_3_O_4_@COF(TBTD-BD)-Au modified by β-CD was synthesized referring to methods that have been previously reported [21]. Detailed synthetic procedures are provided in the Supplementary Information.

### 2.3. Characterization

Transmission electron microscopy (TEM; FEI Tecnai G20 FEI Company, Hillsboro, OR, USA) was conducted to examine the morphologies of composites. Fourier transform infrared (FT-IR) spectra of the prepared materials were recorded using a NEXUS-470 Fourier transform infrared (FT-IR) spectrometer (Thermo Nicolet, Madison, WI, USA). The crystal structure and surface construction of materials were determined by Bruker D8 X-ray diffractometer (with Cu Kα radiation (λ = 0.15418 nm); a scanning rate of 5 deg/min was applied to record the patterns in the 2θ range of 2–80°) and X-ray photoelectron spectroscopy (XPS, Thermo Fisher, Waltham, MA, USA, Escalab 250Xi). The N_2_ adsorption-desorption measurements were conducted at 77 K using an ASAP 2010 micropore physisorption analyzer (Micromeritics, Norcross, GA, USA). MPMS-XL-7 (Quantum Design, San Diego, CA, USA) vibrating sample magnetometer (VSM, Lake Shore 7410, Westerville, OH, USA) readings were taken to examine magnetic properties of the obtained materials. In addition, the thermal stability of materials was evaluated by thermogravimetric analysis (TGA, TA instrument, New Castle, DE, USA, TAQ500).

The adsorption capacity of the adsorbent was determined by using a high-performance liquid chromatography (HPLC)-UV detector. The HPLC analysis was performed on a Baseline C18 column using ACE: water containing 0.1% formic acid (78:22, *v*/*v*) at a flow rate of 1 mL min^−1^ under the UV detection of 260 nm.

### 2.4. Adsorption Experiments

Stock solutions of ACE and THIA (1 mg mL^−1^) were prepared in acetonitrile and diluted with ultrapure water to obtain working solutions immediately before use. For batch adsorption, 10 mg of adsorbent was dispersed in 10 mL of a mixed ACE/THIA solution (50 mg L^−1^) and vortexed for 30 min. The Fe_3_O_4_@COF(TBTD-BD)-Au was then magnetically separated, and the supernatant was filtered through a 0.22 μm membrane. Residual analyte concentrations were determined by HPLC-UV. Unless otherwise stated, experiments used 10 mg adsorbent in 10 mL solution (glass vials), 25 ± 1 °C, and NaCl ionic strength 0.0–0.5 mol L^−1^. Initial pH was adjusted with 1 M formic acid or 1 M ammonia and monitored. All data are mean ± SD (n = 3). HPLC quality control included calibration, blanks, and spike-recovery checks.

The influences of contact time, ionic strength, and pH value on ACE and THIA adsorption were investigated. The initial pH of the solution was adjusted from 3 to 8 using negligible volumes of 1 M formic acid or 1 M ammonia. The effect of contact time was examined over the range of 0–60 min. For ionic strength studies, NaCl was added to the ACE/THIA solution to achieve final concentrations of 0.1–0.5 mol L^−1^. The adsorption capacity for time *t* was calculated using Equation (1):

(1) $$q_{t}=\left(C_{0}-C_{t}\right)V/m$$

The equilibrium analytes adsorption capacity of adsorbent was computed using Equation (2):

(2) $$q_{e}=\left(C_{0}-C_{e}\right)V/m$$

where *C*_0_ (mg L^−1^) and *C_e_* (mg L^−1^) represent the initial and equilibrium concentrations of ACE and THIA, respectively; *V* (L) is the volume of the solution; and *m* (mg) is the mass of Fe_3_O_4_@COF(TBTD-BD)-Au used.

To evaluate the adsorption kinetics, 10 mg of Fe_3_O_4_@COF(TBTD-BD)-Au was dispersed in 10 mL aqueous solutions with initial analyte concentrations of 25, 50, and 100 mg L^−1^. After vortexing for predetermined intervals (1–120 min) at room temperature, the adsorbent was magnetically separated, and the supernatant was filtered and analyzed by HPLC-UV. The adsorption isotherms and thermodynamics were determined by adding 10 mg of Fe_3_O_4_@COF(TBTD-BD)-Au to 10 mL ACE and THIA solution (15–200 mg L^−1^). The mixture was vortexed for 120 min in a water bath at a predetermined temperature (25–45 °C), after which the adsorbent was magnetically separated and the residual ACE and THIA in the supernatant were analyzed by HPLC–UV.

### 2.5. Desorption and Reusability Experiments

To evaluate the desorption ability of the adsorbent, acetonitrile, methanol, or acetone was used as the eluent under ultrasonication for the designated time (5–65 min). For this, the analytes pre-adsorbed Fe_3_O_4_@COF(TBTD-BD)-Au that was first prepared by 10 mg of Fe_3_O_4_@COF(TBTD-BD)-Au, which was dispersed with 10 mL of analytes solution (50 mg L^−1^), and the adsorbent with analytes was collected by a magnet after vortexing at room temperature for 30 min. After this desorption stage, the supernatant was filtered and the filtrate was analyzed with HPLC.

To study the reusability of Fe_3_O_4_@COF(TBTD-BD)-Au for analytes, the used Fe_3_O_4_@COF(TBTD-BD)-Au was washed with 3 mL acetonitrile three times and dried for another adsorption cycle (repeated 6 times).

## 3. Result and Discussion

### 3.1. Preparation and Characterization

**Figure 2:**
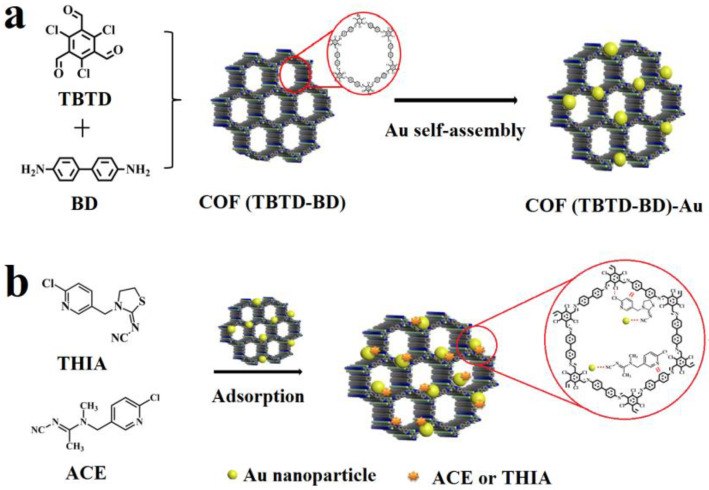
(**a**): Schematic illustration of the synthesis of COF (TBTD-BD)-Au and inset with chemical structure of the COF pore; (**b**): the chemical structures of analytes and the removal mechanism of ACE and THIA on Fe_3_O_4_@COF(TBTD-BD)-Au.

**Figure 3:**
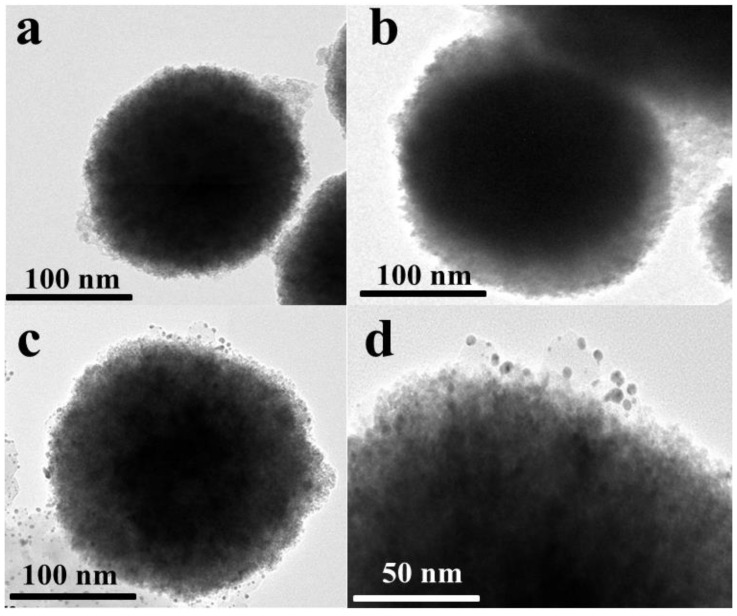
TEM images of (**a**): Fe_3_O_4_, (**b**): Fe_3_O_4_@COF(TBTD-BD), (**c**,**d**): Fe_3_O_4_@COF(TBTD-BD)-Au.

The COF was synthesized through a Schiff base reaction between TBTD and BD in DMSO, with acetic acid as the catalyst at room temperature. Subsequently, Au atoms were incorporated via self-assembly. The structure of COF(TBTD-BD) is depicted in Figure 2a. The morphological structure of Fe_3_O_4_@COF(TBTD-BD)-Au was characterized by TEM. Figure 3a reveals nearly spherical pure Fe_3_O_4_ particles with an average size of 200 nm.

As shown in Figure 3b, a thin layer of COF(TBTD-BD) coats the surface of the dark magnetite particles, forming a core-shell structure. Figure 3c,d show that Au nanoparticles are dispersed on the surface of the Fe_3_O_4_@COF(TBTD-BD) microspheres.

**Figure 4:**
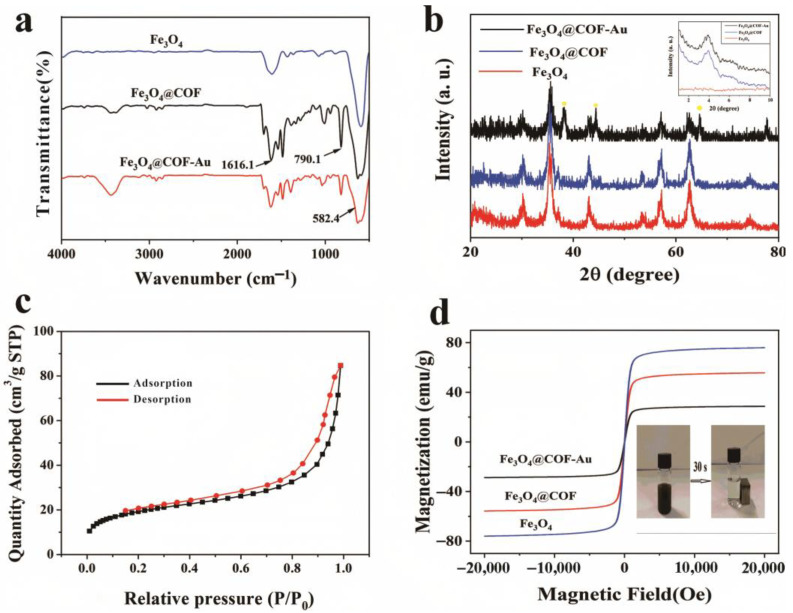
(**a**): FT-IR spectra of Fe_3_O_4_, Fe_3_O_4_@COF(TBTD-BD), and Fe_3_O_4_@COF(TBTD-BD)-Au; (**b**): XRD of Fe_3_O_4_, Fe_3_O_4_@COF(TBTD-BD), and Fe_3_O_4_@COF(TBTD-BD)-Au; (**c**): N_2_ adsorption-desorption isotherms of Fe_3_O_4_@COF(TBTD-BD)-Au; (**d**): magnetic hysteresis curve of Fe_3_O_4_, Fe_3_O_4_@COF(TBTD-BD), and Fe_3_O_4_@COF(TBTD-BD)-Au.

The FT-IR spectra (shown in Figure 4a) identified the characteristic functional groups of Fe_3_O_4_, Fe_3_O_4_@COF(TBTD-BD), and Fe_3_O_4_@COF(TBTD-BD)-Au. A peak at 582.4 cm^−1^, observed in all spectra of the magnetic materials, indicates Fe-O vibration, confirming the presence of Fe_3_O_4_ nanoparticles. Additionally, a new adsorption band at 1616.1 cm^−1^ represents the C=N band (Figure S1) and a peak at 790.1 cm^−1^ signifies the C-Cl bond, which confirms the successful synthesis of COF(TBTD-BD) [22]. The residual band near 1700 cm^−1^ is also present in the Fe_3_O_4_@COF spectrum, which could arise from either a minor fraction of unreacted aldehyde groups or an overlap with C=O-like vibrations from COF-framework defects.

Moreover, the XPS spectrum of Fe_3_O_4_@COF(TBTD-BD)-Au, shown in Figure S5a, exhibits distinct Au 4f and Cl 2p peaks, further corroborating the successful preparation of the material. The crystallinity of the synthesized material was characterized by XRD. Figure 4b shows the diffraction peaks at 30.16°, 35.35°, 37.1°, 42.9°, 57.1°, and 62.57°, which correspond to the (220), (311), (222), (400), (511), and (440) planes of Fe_3_O_4_ and align well with the standard JCPDS 19-0629 Fe_3_O_4_ data [23]. Additionally, three peaks at 38.2°, 44.4°, and 64.6° can be indexed to (111), (200), and (220) diffraction of Au (JCPDS 04-0784) [24]. The small peak at 2θ = 3.9° (shown in Figure 4b and Figure S2) signifies small-angle diffraction from COF, indicating the successful synthesis of COF. These results confirmed the successful creation of Fe_3_O_4_@COF(TBTD-BD)-Au. The N_2_ adsorption-desorption isotherms, displayed in Figure 4c, indicated a Brunauer–Emmett–Teller (BET) surface area and pore volume of Fe_3_O_4_@COF(TBTD-BD)-Au of 84.68 m^2^ g^−1^ and 0.065 cm^3^ g^−1^, respectively. The relatively modest BET surface area may result from chloride counterions obstructing some network pores [25]. Although the BET area is modest, selective uptake is governed by site-specific interactions (Au–nitrile; Cl-decorated microenvironment) rather than the total surface area.

The curves of field-dependent magnetization for Fe_3_O_4_, Fe_3_O_4_@COF(TBTD-BD), and Fe_3_O_4_@COF(TBTD-BD)-Au are shown in Figure 4d. The magnetization curves of Fe_3_O_4_, Fe_3_O_4_@COF(TBTD-BD), and Fe_3_O_4_@COF(TBTD-BD)-Au exhibit typical sigmoidal shapes, which is indicative of their superparamagnetic behavior. The saturated magnetization values of the initial Fe_3_O_4_, Fe_3_O_4_@COF(TBTD-BD), and Fe_3_O_4_@COF(TBTD-BD)-Au are 75.9, 55.72, and 28.76 emu/g, respectively. Despite the decrease in saturated magnetization values, they remain sufficiently strong for separation using an external magnet. The inset of Figure 4d illustrates that the finely dispersed Fe_3_O_4_@COF(TBTD-BD)-Au can be efficiently separated from the solution under an externally magnetic field within 30 s, and that the recovery yield after separation can reach 99–100%. The thermogravimetric curve indicates that Fe_3_O_4_@COF(TBTD-BD)-Au exhibits no significant weight loss upon heating from 0 to 400 °C (Figure S3), which demonstrates its high thermal stability.

### 3.2. Effects of pH, Ionic Strength and Contact Time

**Figure 5:**
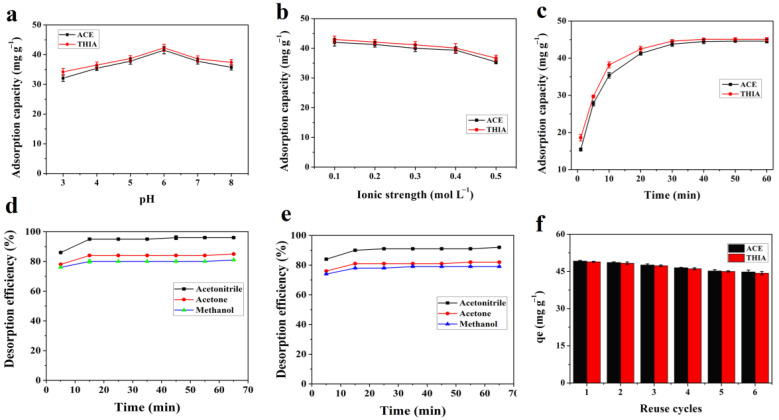
Effect of (**a**): pH, (**b**): ionic strength, (**c**): contact time, (**d**): desorption time and desorption solution for ACE, (**e**): desorption time and desorption solution for THIA, and (**f**): reuse cycles on desorption of analytes from Fe_3_O_4_@COF(TBTD-BD)-Au.

The effect of the solution pH on ACE and THIA removal was systematically investigated over the range of pH 3–8. As depicted in Figure 5a, it is evident that the adsorption capacities of ACE and THIA exhibit pronounced peaks at a pH of 6. Below a pH of 5, the adsorption capacity experiences a notable decline. This phenomenon can be attributed to the rapid aggregation of gold nanoparticles induced by the positively charged citrate ions, which makes it challenging for them to interact effectively with the cyano groups of ACE and THIA. Furthermore, the adsorption capacity diminishes when the pH values exceed 7.5, likely due to the hydrolysis of cyano groups under basic conditions, resulting in the reduced ability of Au nanoparticles to bind ACE and THIA. Consequently, a pH of 6.0 was selected as the optimal condition for the entirety of this study. The impact of the NaCl concentration on the removal of ACE and THIA was systematically examined due to the presence of salts in water, which can lead to elevated ionic strength and potentially influence the adsorption process. Figure 5b shows that the adsorption capacity of ACE and THIA on Fe_3_O_4_@COF(TBTD-BD)-Au remains nearly constant at NaCl concentrations below 0.2 mol L^−1^, which indicates strong resistance to ionic interference. However, when the NaCl concentration exceeds 0.2 mol L^−1^, the adsorption capacities decrease, likely because higher ionic strength increases the solution viscosity and matrix effects and thereby reduces the diffusion rate of analytes and hinders their adsorption on Fe_3_O_4_@COF(TBTD-BD)-Au [26]. A good dispersion of adsorbent is advantageous in enhancing the adsorption of ACE and THIA. In this experiment, the effect of contact time was investigated in the range of 2–60 min. Figure 5c demonstrates that about 84–90% of the equilibrium adsorption capacity is achieved within a time close to 20 min and then it exhibits small increases in the following 30 min.

### 3.3. Adsorption Kinetics

**Figure 6:**
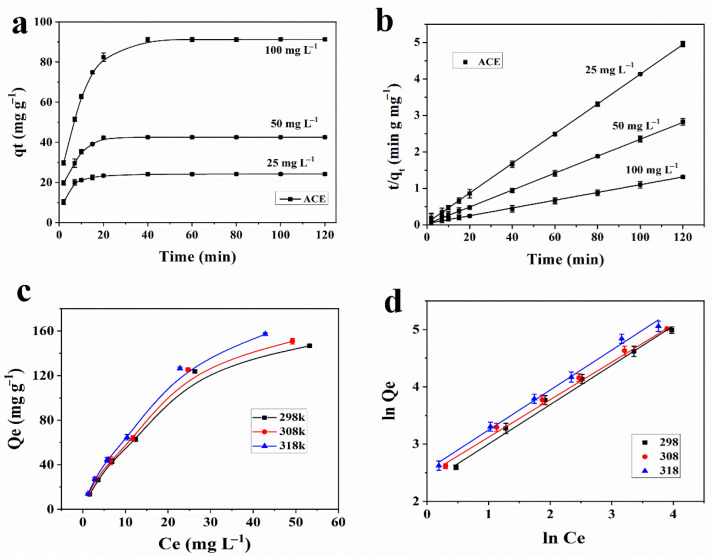
(**a**): Time-dependent adsorption capacity; (**b**): pseudo-second-order kinetics plots; (**c**): adsorption isotherms; and (**d**): Freundlich plots for the adsorption of ACE on Fe_3_O_4_@COF(TBTD-BD)-Au.

The adsorption kinetics of ACE and THIA on Fe_3_O_4_@COF(TBTD-BD)-Au were investigated at three initial concentrations (25, 50, and 100 mg L^−1^). The adsorption of ACE and THIA onto Fe_3_O_4_@COF(TBTD-BD)-Au was observed to be time-dependent, as seen in Figure 6a and Figure S4a. Typically, 92 to 95% of the ultimate adsorption occurs within the initial 20 min of contact. In addition, the sorption rate is notably high during the initial phase, and the adsorption rate of ACE and THIA increases with higher initial concentrations.

The adsorption performance of Fe_3_O_4_@COF(TBTD-BD)-Au was evaluated using pseudo-first-order and pseudo-second-order kinetic models. The pseudo-first-order kinetic equation is expressed as follows, where *q_e_* and *q_t_* (mg g^−1^) denote the adsorption capacities at equilibrium and at time *t* (min), respectively, and *k*_1_ (min^−1^) is the rate constant of the pseudo-first-order model.

(3) $$\ln\left(q_{e}-q_{t}\right)=\ln q_{e}-k_{1}t$$

The pseudo-second-order kinetics equation is given below:

(4) $$t/q_{t}=1/K_{2}q_{e}^{2}+t/q_{e}$$

where *k*_2_ is the rate constant of pseudo-second-order adsorption (g mg^−1^ min^−1^).

The low R^2^ values shown in Table S1 clearly indicated the poor fit of the pseudo-first-order model for ACE and THIA on Fe_3_O_4_@COF(TBTD-BD)-Au across the entire treatment period. Plots of *t*/*q_t_* versus t at three initial concentrations are presented in Figure 6b and Figure S4b. The kinetic parameters of the pseudo-second-order model, including the rate constant (*k*_2_), equilibrium adsorption capacity (*q_e_*), and regression coefficients, are summarized in Table 1. The results show that the pseudo-second-order model fits the experimental data quite well, as the R^2^ values are greater than 0.99. This indicates the applicability of the second-order kinetic model to describe the adsorption process of ACE and THIA onto Fe_3_O_4_@COF(TBTD-BD)-Au. Furthermore, the k_2_ values decreased with increasing initial concentrations of ACE and THIA, suggesting that the adsorption process depends on both the adsorbate and adsorbent [27], which in turn suggests that strong interactions occurred between the analytes and adsorbent.

### 3.4. Adsorption Isotherms and Thermodynamics

Adsorption isotherms were investigated at three temperatures (298, 308, and 318 K). The Langmuir and Freundlich models (Equations (5) and (6)) were applied to interpret the adsorption process. The Langmuir model describes monolayer adsorption on homogeneous surfaces [28], whereas the Freundlich model accounts for adsorption on heterogeneous surfaces with non-uniform energy distributions [29]. The equations are given as follows:

(5) $$C_{e}-q_{e}=C_{e}/q_{m}+1/q_{m}b$$

(6) $$\ln q_{e}=\ln K_{f}+\ln C_{e}/n$$

where *C_e_* (mg L^−1^) is the equilibrium concentration of ACE and THIA, *b* (L mg^−1^) is the Langmuir constant, and *q_m_* (mg g^−1^) represents the maximum adsorption capacity (mg g^−1^). *n* and *K_f_* are Freundlich constants that correspond to the adsorption intensity and adsorption capacity, respectively.

Compared with the Langmuir model, the Freundlich model is a better fit for the adsorption process of ACE and THIA on Fe_3_O_4_@COF(TBTD-BD)-Au (Figure 6d and Figure S4d). The maximum adsorption capacity of ACE and THIA is increased when the adsorption temperature is increased from 298 K to 318 K, which indicates that the adsorption of ACE and THIA on Fe_3_O_4_@COF(TBTD-BD)-Au is endothermic (Figure 6c and Figure S4c). The Freundlich model indicates that the adsorption of ACE and THIA onto Fe_3_O_4_@COF(TBTD-BD)-Au is likely to involve interactions between the adsorbents and analytes [30]. The Freundlich sorption coefficients *K_f_* and n are empirical constants representing the extent of sorption and the degree of non-linearity of sorption, respectively. The values of 1/*n* listed in Table 2 are significantly lower than 1, confirming that the adsorption of ACE and THIA occurs easily [31].

The thermodynamic parameters were calculated to study the adsorption mechanism for ACE and THIA on Fe_3_O_4_@COFs (TBTD-BD)-Au. The equations are as follows:

(7) $$\Delta G=-RT\ln q_{e}/C_{e}$$

(8) $$\ln q_{e}/C_{e}=\Delta S/R-\Delta H/(RT)$$

where *R* is the universal gas constant (8.314 J mol^−1^ K^−1^). *K*_0_ was calculated from the intercept of the plot of ln(*q_e_*/*C_e_*) versus *q_e_*. The thermodynamic parameters Δ*S* and Δ*H* were derived from the slope and intercept of the plot of ln *K*_0_ versus 1/*T* (Equation (8)), and the calculated values are summarized in Table S2. The negative Δ*G* values confirm that the adsorption of ACE and THIA on Fe_3_O_4_@COF(TBTD-BD)-Au is spontaneous. The positive enthalpy change (Δ*H*) confirms the endothermic nature of the adsorption process, which is consistent with the observed enhancement in adsorption capacity at elevated temperatures. The positive Δ*S* indicates the augmented randomness during the process.

### 3.5. Desorption and Reusability

From a sustainability perspective, facile regeneration is essential for adsorbent reuse. Methanol, acetonitrile, and acetone were tested as desorption solvents for ACE and THIA from Fe_3_O_4_@COF(TBTD-BD)-Au (Figure 5d,e). Among them, acetonitrile exhibited the highest desorption efficiency, achieving complete desorption under ultrasonication within 15 min. After one regeneration cycle, the mass loss of the adsorbent was less than 5%. Moreover, the adsorption capacity of Fe_3_O_4_@COF(TBTD-BD)-Au toward ACE and THIA decreased by only 8.5% after six adsorption–desorption cycles (Figure 5f), which demonstrates its excellent reusability. To demonstrate the advantages of the proposed adsorbent, the comparisons with the previously reported adsorbents are shown in Table 3. The results show that our adsorbent exhibits a higher adsorption capacity and shorter time-using, further indicating the Fe_3_O_4_@COF(TBTD-BD)-Au is feasible for ACE and THIA removal.

### 3.6. Removal Selectivity for ACE and THIA

**Figure 7:**
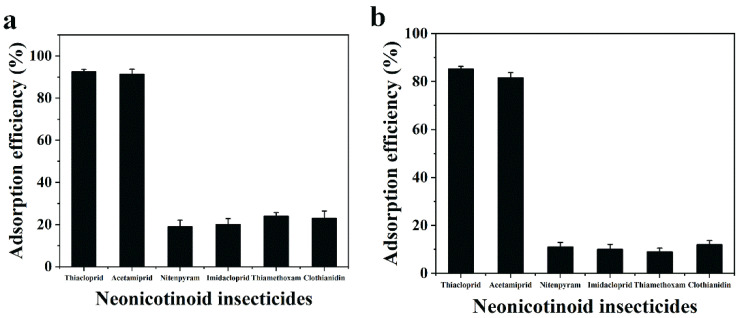
(**a**): Adsorption ability of single type neonicotinoid insecticide; (**b**): the competitive test for six neonicotinoid insecticide (THIA, ACE, nitenpyram, imidacloprid, thiamethoxam, and clothianidin) on Fe_3_O_4_@COF(TBTD-BD)-Au.

The adsorption selectivities of Fe_3_O_4_@COF(TBTD-BD)-Au for ACE and THIA were studied. Firstly, the removal ability of adsorbent for the single pesticide with concentration of 50 mg L^−1^ was investigated. Figure 7a shows that the adsorption efficiency of THIA is 92.5%, followed by ACE with 91.3%, which is higher than that for other neonicotinoids, indicating the high removal efficiency of Fe_3_O_4_@COF(TBTD-BD)-Au for ACE and THIA. To further verify the high adsorption selectivity of Fe_3_O_4_@COF(TBTD-BD)-Au, a competitive test was conducted. A mixed solution of these neonicotinoids including NIT, THI, IMI, CLO, ACE, and THIA was tested. The results are presented in Figure 7b. In the competitive test, the adsorption efficiency of THIA and ACE was 85.2% and 81.6%, respectively, which is higher than that for other co-existing analytes. The competitive test results further verify the high selectivity of Fe_3_O_4_@COF(TBTD-BD)-Au for ACE and THIA.

### 3.7. Removal Mechanism

Given the remarkable adsorption performance of Fe_3_O_4_@COF(TBTD-BD)-Au toward ACE and THIA, it is essential to elucidate the underlying adsorption mechanism. Based on the structures of analytes and Fe_3_O_4_@COF(TBTD-BD)-Au, we can speculate that the interaction of the cyano group of analytes with Au nanoparticles, hydrogen bonding (Cl-H…Cl), and π–π interaction may play significant roles during the adsorption. Thus, XPS characterization of Fe_3_O_4_@COF(TBTD-BD)-Au after adsorption, influencing factors, adsorption kinetics, and adsorption isotherms were combined to investigate the removal mechanism.

**Figure 8:**
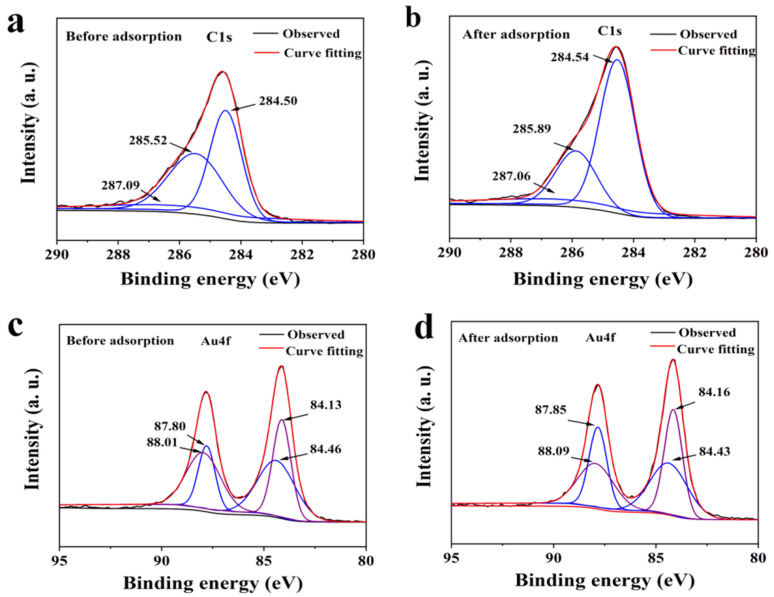
(**a**,**b**): XPS spectra of C1s before and after adsorption; (**c**,**d**): XPS spectra of Au 4f before and after adsorption.

As illustrated in Figure 8a,b, the C=C component of the C 1s XPS spectrum shifts from 284.50 eV to 284.54 eV upon adsorption, which is indicative of π–π stacking interactions between the adsorbates and the conjugated framework of Fe_3_O_4_@COF(TBTD-BD)-Au [32]. The cyano group is a kind of soft base, which can form strong complexes with low-valence heavy metals of soft acids. Figure 8c,d show that the peaks of Au 4f at 84.13 eV, 84.46 eV, 87.80 eV, and 88.01 eV were shifted to 84.16 eV, 84.43 eV, 87.85 eV, and 88.09 eV, respectively, after ACE and THIA adsorption, which revealed the formation of interaction between the Au and cyano group. The stability constant of the complex formed by gold and the cyano group is 38.3, higher than that of other metal ions (Ag^+^, Cd^2+^, Fe^2+^ and Ni^2+^). The interaction between the cyano group and Au nanoparticles can be explained by Equation (9).

(9) $$4\mathrm{Au}+8{\mathrm{CN}}^{-}+2H_{2}O+O_{2}=4\mathrm{Au}{\left(\mathrm{CN}\right)}_{2}+4OH^{-}$$

The Cl 2p spectra of Fe_3_O_4_@COF(TBTD-BD)-Au contained peaks at 200.64 eV and 202.29 eV, which shifted to 200.73 eV and 202.34 eV after ACE and THIA adsorption (Figure S5c,d). In addition, the intensity of C-Cl spectrums was decreased after adsorption, possibly caused by the formation of the hydrogen bindings (Cl-H…Cl). In addition, the adsorption capacities of ACE and THIA on Fe_3_O_4_@COF(TPBD), Fe_3_O_4_@COF(TBTD-BD), Fe_3_O_4_@COF(TBTD-BD)-Au, and Fe_3_O_4_@COF(TBTD-BD)-Au-β-CD were compared (Figure S5b). The results indicate that the maximum adsorption capacity is achieved when Fe_3_O_4_@COF(TBTD-BD)-Au us used as the adsorbent. It is worth noting that the adsorption capacity of the material would reduce if the active sites of the Au nanoparticles were masked through a common hydrophilic substance such as β-cyclodextrin. These results reveal the importance of Au nanoparticles in ACE and THIA removal. In addition, the Fe_3_O_4_@COF(TBTD-BD) has a higher ACE and THIA capture ability than Fe_3_O_4_@COF(TPBD), which illustrates the affinity function between Cl-H…Cl and also improves its adsorption performance. Our kinetic data are well described by the pseudo-second-order model, which indicates an apparent second-order dependence on the fraction of unoccupied sites. Because the pseudo-second-order model equation is empirical and can arise from different rate-limiting steps, we do not infer the adsorption mechanism solely from this fit. The adsorption isotherms show that a high temperature is beneficial for the adsorption process, and adsorption occurred spontaneously. To diagnose the rate control, Weber–Morris plots were included (Supplementary Figure S6). The plots show two segments and non-zero intercepts across 25–100 mg L^−1^, indicating mixed control. External film diffusion plus rapid surface binding at the designed Au/COF sites dominates the initial stage, whereas intraparticle diffusion governs the later stage. This aligns with the rapid approach to equilibrium (~20 min) reported in the main text.

**Table 3:** Comparison of the proposed adsorbent with other adsorbents for the extraction of neonicotinoid insecticides.

Adsorbents	Adsorbate	Equilibrium Time	q_m_ (mg/g)	References
IPP-MMIPs ^a^	Paichongding	150 min	13.30	[33]
AMCCP ^b^	ACE	100 min	142.36	[34]
Fe_3_O_4_/ZIF-67/GO	ACE and THIA	50 min	-	[35]
Carbon cryogel	Imidacloprid	24 h	80–100	[36]
Fe_3_O_4_@COF(TBTD-BD)-Au	ACE and THIA	40 min	157	This work
GCA ^c^ (F-200)	Imidacloprid, Clothianidin, Thiamethoxam and Thiacloprid	20 d	60–150	[37]

^a^: Paichongding-magnetic molecularly imprinted nanoparticles. ^b^: Activated modified carbon-based porous particle. ^c^: Granular active carbon.

### 3.8. Adsorption Performance in Beverages

The adsorption of ACE and THIA in beverage samples (orange juice and cola drink) was evaluated to demonstrate the practical feasibility of using Fe_3_O_4_@COF(TBTD-BD) for target removal. It is worth noting that the samples we selected are the intermediate products of the beverage production line, in order to minimize the impact on the taste of the finished product and reduce the interference of additives in the beverage with the removal efficiency. Recoveries (R) were determined on the basis of Equation (10):

(10) $$R\%=\frac{C_{\mathrm{spiked}}-C_{\mathrm{matrix}}}{C_{\mathrm{standard}}}\times 100\%$$

in which C_spiked_, C_matrix_, and C_standard_ represent concentrations of spiked and unspiked samples and standard solution, respectively. As shown in Figure S7, the adsorption recovery was higher than 95.8%, which indicates the great potential of novel Fe_3_O_4_@COF(TBTD-BD) for ACE and THIA adsorption and removal in real beverages samples.

## 4. Conclusions

In summary, we have designed and synthesized a tailor-made COF designated as Fe_3_O_4_@COF(TBTD-BD)-Au for highly selective removal of ACE and THIA from beverages. By judicious design of a COF ligand and post-functionalized group, the adsorption capacity of a novel material for ACE and THIA has significantly improved. Depending on the strong affinity function between the cyano groups and Au nanoparticles, hydrogen bonding (Cl-H…Cl), and π–π interaction, a maximum adsorption capacity of 157 mg g^−1^ was achieved. The rapid adsorption kinetics, large adsorption capacity, and good reusability give Fe_3_O_4_@COF(TBTD-BD)-Au high potential in ACE and THIA removal from other matrixes. This work also revealed the feasibility of the rational design and synthesis of functionalized COFs for use as novel adsorbents for the highly selective and efficient removal of targeted analytes from complex matrixes. However, further improvements are still required: (1) the solvents currently used for regeneration are volatile organic compounds and not environmentally friendly, and thus greener alternatives such as bio-based solvents should be evaluated in future studies; (2) potential food-safety concerns must be carefully addressed, as traces of adsorbent or nanoparticle components could theoretically remain in beverages, so thorough toxicological assessment and safety validation are necessary.

## Figures and Tables

**Table 1:** Kinetic parameters of pseudo-second-order kinetic model for the adsorption of ACE and THIA.

Analytes	C_0_ (mg L^−1^)	Pseudo-Second-Order Kinetic Model		
		q_e_ (mg g^−1^)	K_2_	R^2^
ACE	25	24.2	0.026	0.9908
	50	42.7	0.013	0.9916
	100	91.3	0.0027	0.9932
THIA	25	24.9	0.028	0.9917
	50	44.3	0.014	0.9926
	100	92.9	0.0026	0.9955

**Table 2:** Parameters of adsorption isotherms of ACE and THIA onto Fe_3_O_4_@COF(TBTD-BD)-Au at different temperatures.

Analytes	Temperature	Langmuir Model			Freundlich Model		
		*q_m_* (mg g^−1^)	*b* (L mg^−1^)	*R* ^2^	*K_f_*	1/*n*	*R* ^2^
ACE	298 K	146.8	0.5978	0.8109	1.1858	0.6882	0.9985
	308 K	150.8	0.5961	0.8202	1.3972	0.7086	0.9982
	318 K	157.2	0.6002	0.8314	1.5547	0.7144	0.9980
THIA	298 K	149.9	0.6243	0.8331	1.0422	0.6610	0.9982
	308 K	151.3	0.5981	0.8209	1.4353	0.7131	0.9964
	318 K	156.1	0.5859	0.8241	1.7423	0.7292	0.9988

## Data Availability

The original contributions presented in this study are included in the article/Supplementary Materials. Further inquiries can be directed to the corresponding authors.

## Supplementary Materials

The following supporting information can be downloaded at: https://www.mdpi.com/article/10.3390/nano15201596/s1, Figure S1. FT-IR spectra of Fe_3_O_4_@COF(TBTD-BD), TBTD and BD. Figure S2. The XRD patterns of Fe_3_O_4_@COF(TBTD-BD). Figure S3. TGA curve of Fe_3_O_4_@COF(TBTD-BD)-Au. Figure S4. a: Time-dependent adsorption capacity; b: pseudo-second-order kinetics plots; c: adsorption isotherms and d: Freundlich plots for the adsorption of THIA on Fe_3_O_4_@COF(TBTD-BD)-Au. Figure S5. a: XPS survey spectrum of Fe_3_O_4_@COF(TBTD-BD)-Au; b: the comparison of adsorption capacity for different materials; c and d: XPS spectra of Cl2p before and after adsorption. Figure S6. Weber-Morris diagnostics for ACE and THIA adsorption on Fe_3_O_4_@COF(TBTD-BD)Au. Plots of q_t_ versus t^1/2^ (min^0.5^) for (a) ACE and (b) THIA at three initial concentrations (25, 50, and100 mg L^−1^). Figure S7. Adsorption of spiked ACE and THIA (50 mg L^−1^) from origin juice and cola drink samples on Fe_3_O_4_@COF(TBTD-BD)-Au. Table S1. Kinetic Parameters of pseudo-first-order kinetic model for the adsorption of ACE and THIA. Table S2. Analysis of adsorption thermodynamics of Fe_3_O_4_@COF(TBTD-BD)-Au towards ACE and THIA with different initial concentrations.
