# Supplementary material for: Unique Design of Functionalized Covalent Organic Frameworks for Highly Selective Removal of Cyano-Neonicotinoids
Source: Nanomaterials (Basel). 2025 Oct 20;15(20):1596. doi: 10.3390/nano15201596 (PMC12566725; doi:10.3390/nano15201596)
Supplement: Supplementary file 1 [file nanomaterials-15-01596-s001.zip › nanomaterials-3833704-supplementary.pdf]

Supplementary information

Unique Design of Functionalized Covalent Organic Frameworks for Highly Selective Removal of Cyano-Neonicotinoids

Yan Yang *, Shuojie Wang, Wenxin Mai, Shiyu Wei, Guixiang Teng, Peng Pu, Jiaxing Zhao and Yongqiang Tian *

School of Biological and Pharmaceutical Engineering, Lanzhou Jiaotong University, Lanzhou 730070, China; wsjlzjt@163.com (S.W.); 18820610921@163.com (W.M.); wsy@lzjtu.edu.cn (S.W.); tenggx@lzjtu.edu.cn (G.T.); pupeng@lzjtu.edu.cn (P.P.); zhaojiaxing@lzjtu.edu.cn (J.Z.)

* Correspondence: yangy@lzjtu.edu.cn (Y.Y.); tianyq@mail.lzjtu.cn (Y.T.)

Abbreviations

Acetamiprid	ACE
Thiacloprid	THIA
Covalent organic frameworks	COF
2,4,6-trichloro-benzene-1,3,5-tricarbaldehyde	TBTD
benzidine	BD
thiamethoxam	THI
imidacloprid	IMI
clothianidin	CLO
nitenpyram	NIT
1,3,5-triformylphloroglucinol	TP

Experimental

The Fe_3O_4@COF(TPBD) was synthesized according to the methods that have been previously reported. First, 16 mg of Fe_3O_4 and 16 mg of BD were added to 11 mL THF with ultrasound for 30 min and then refluxed with stirring for 30 min. Next, 4 mL of THF solution containing 12 mg of TP was added to the above mixture. The reaction was allowed to proceed under mechanical stirring at 50 °C. After 3 h, the brown particles were magnetically separated and washed with MeOH for four times. Finally, the product was dried under vacuum at 45 °C.

The Fe_3O_4@COF(TBTD-BD)-Au-β-CD was synthesized followed methods that have been previously reported. Typically, β-CD (40 mg) was dissolved in water (10 mL) and sonicated for 15 minutes. Next, the mixture was added to 60 mg of Fe_3O_4@COF@Au and stirred at room temperature for 24 hours to obtain a black

precipitate. After elution with water until the supernatant was colorless, it was dried in a 45 ºC oven for 12 hours for further use.

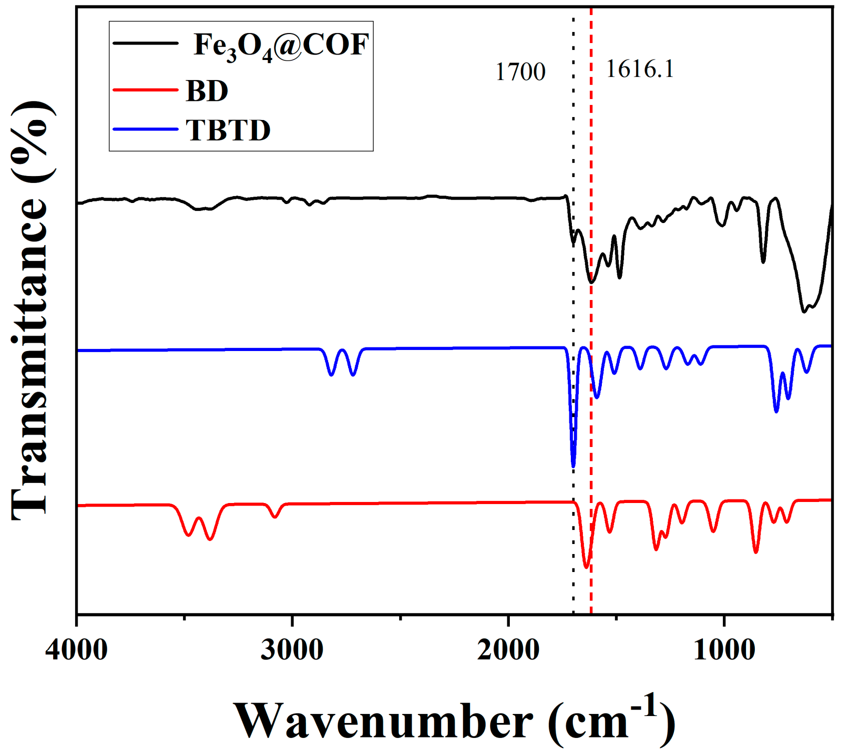

Figure S1. FT-IR spectra of Fe_3O_4@COF(TBTD-BD), TBTD and BD.

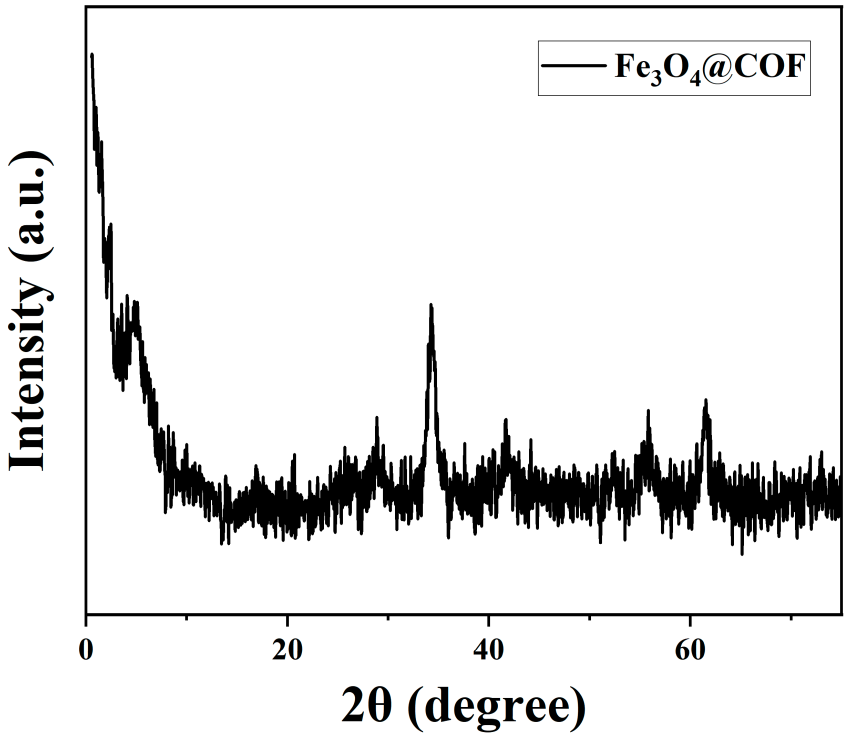

Figure S2. The XRD patterns of Fe_3O_4@COF(TBTD-BD).

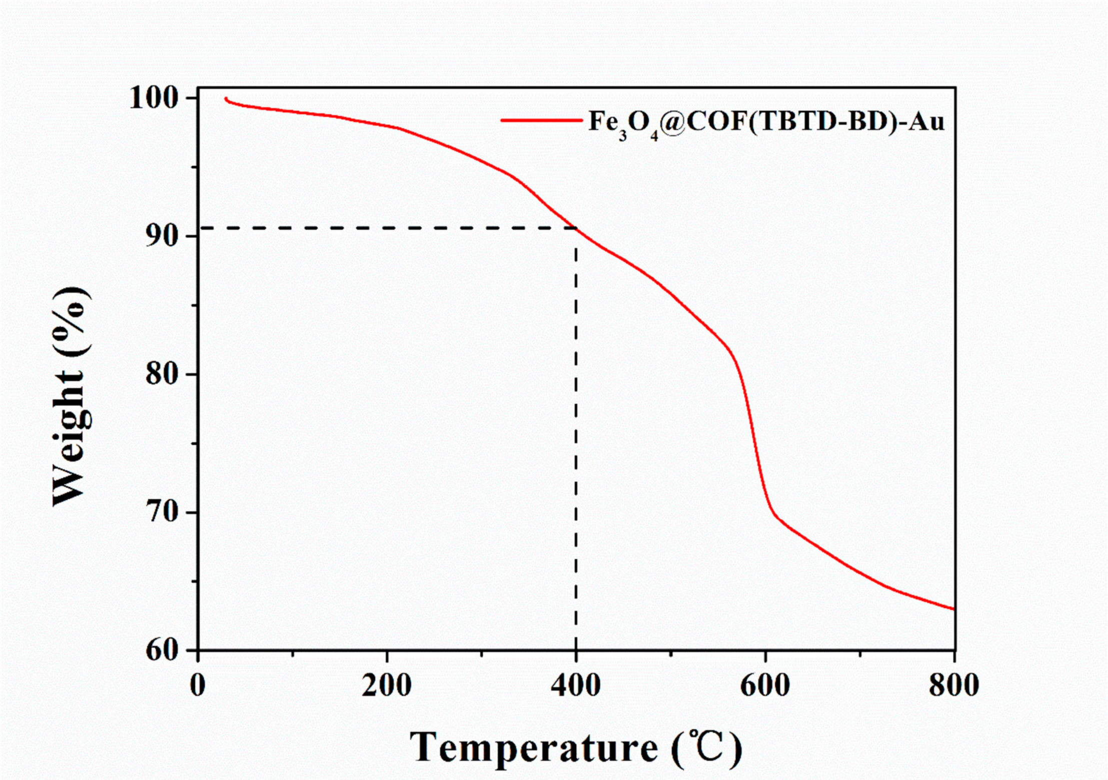

Figure S3. TGA curve of Fe_3O_4@COF(TBTD-BD)-Au.

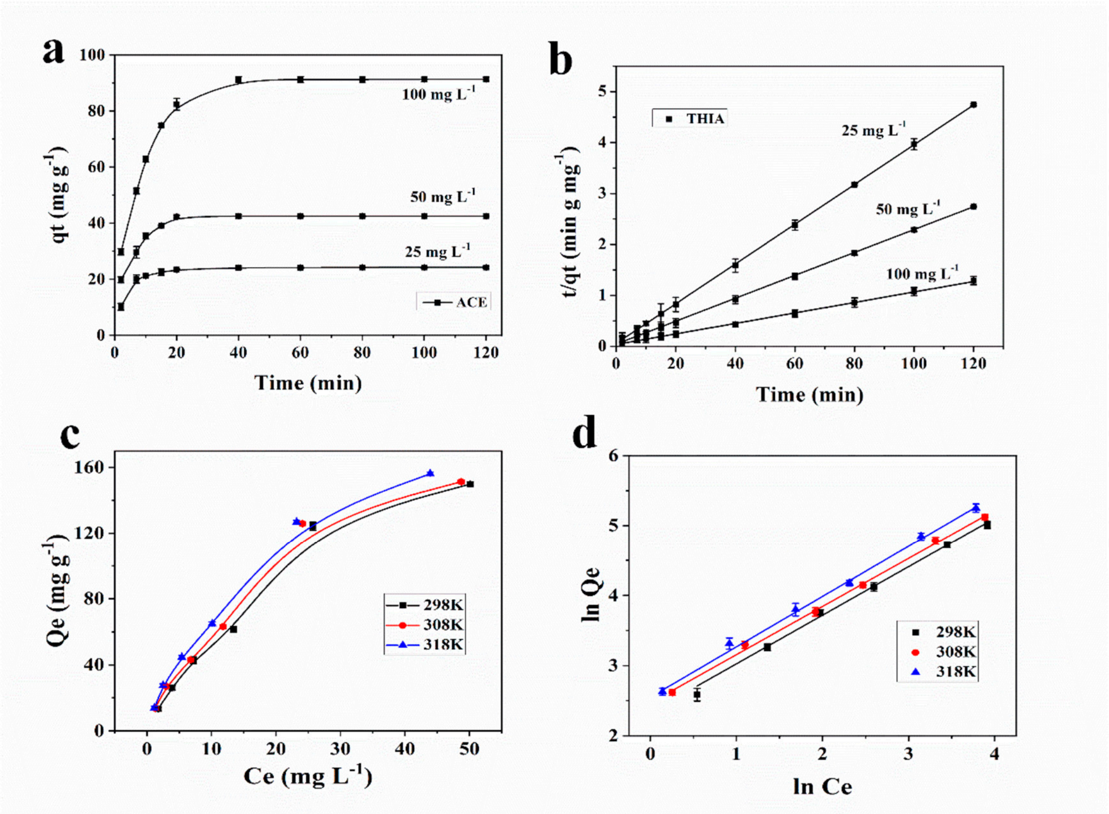

Figure S4. a: Time-dependent adsorption capacity; b: pseudo-second-order kinetics plots; c: adsorption isotherms and d: Freundlich plots for the adsorption of THIA on Fe_3O_4@COF(TBTD-BD)-Au.

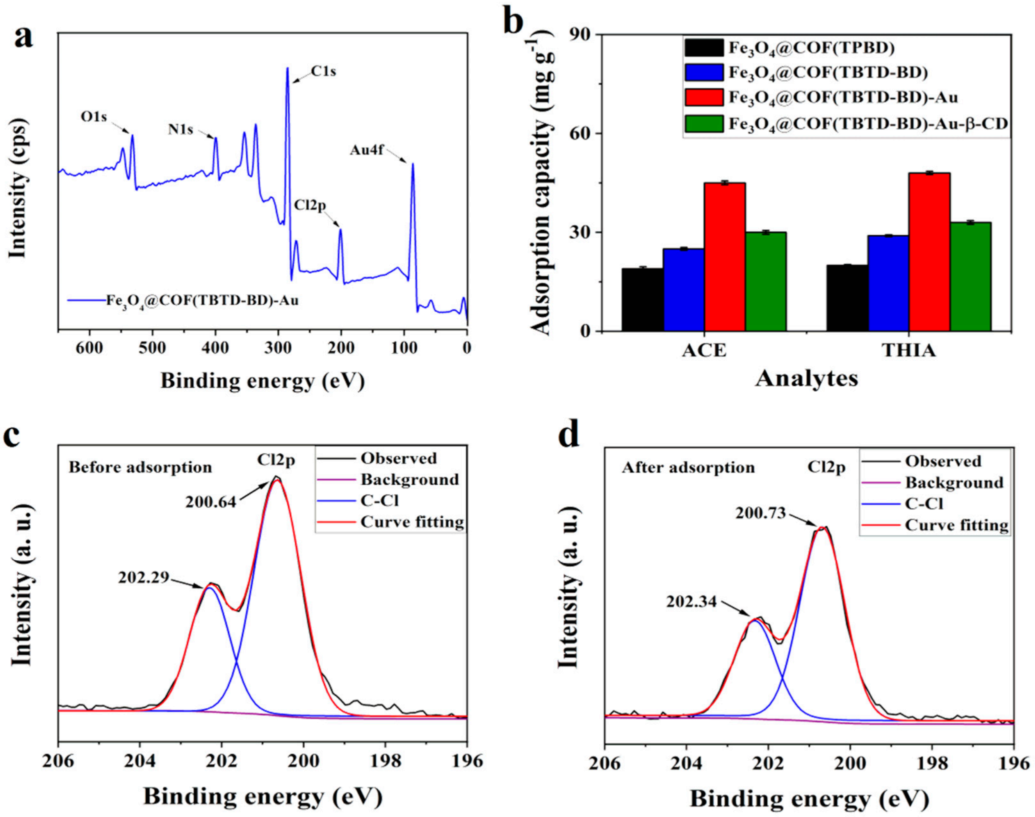

Figure S5. a: XPS survey spectrum of Fe_3O_4@COF(TBTD-BD)-Au; b: the comparison of adsorption capacity for different materials; c and d: XPS spectra of Cl2p before and after adsorption.

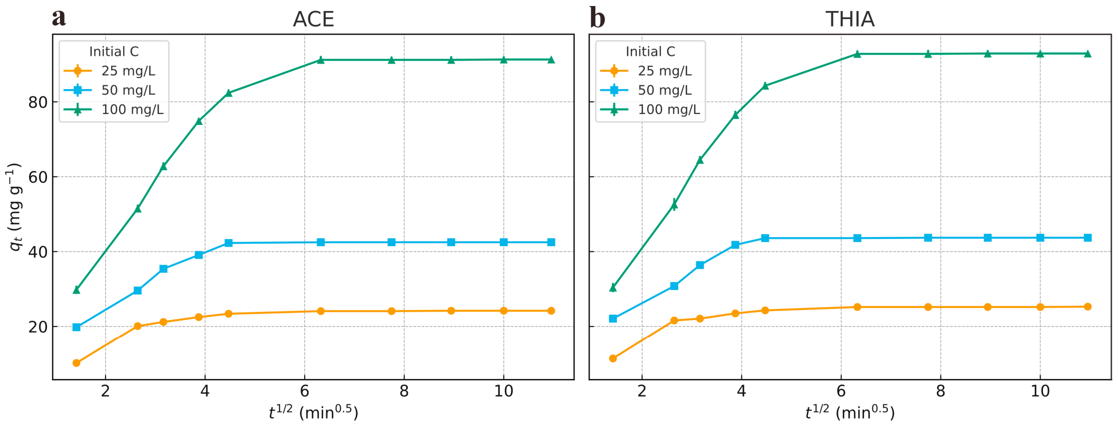

Figure S6. Weber-Morris diagnostics for ACE and THIA adsorption on Fe_3O_4@COF(TBTD-BD)Au. Plots of q_t versus $t^{1/2}$ ($min^{0.5}$) for (a) ACE and (b) THIA at three initial concentrations (25, 50, and100 mg L^{-1}).

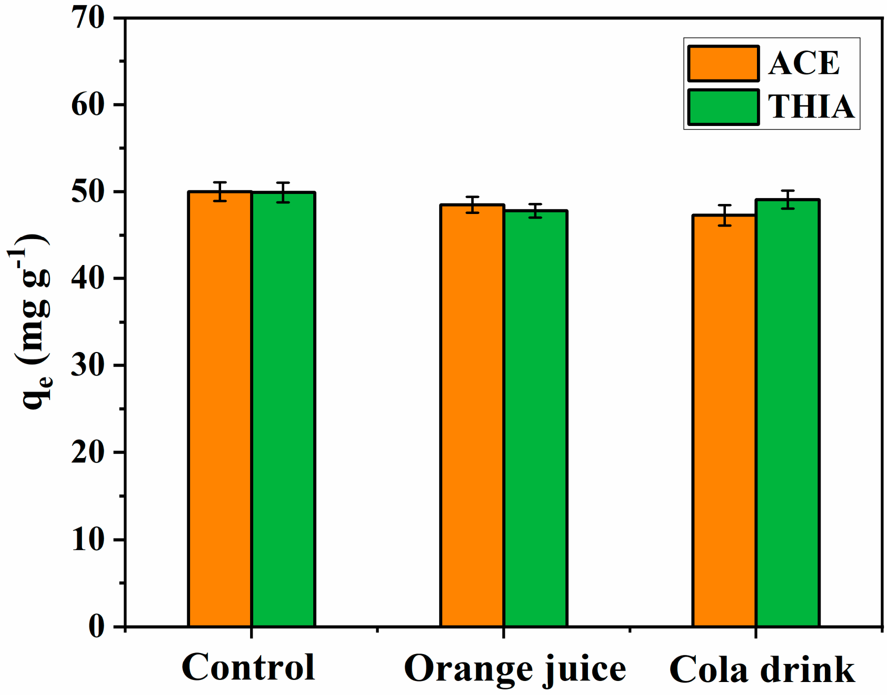

Figure S7. Adsorption of spiked ACE and THIA (50 mg L^{-1}) from origin juice and cola drink samples on Fe_3O_4@COF(TBTD-BD)-Au.

Table S1. Kinetic Parameters of pseudo-first-order kinetic model for the adsorption of ACE and THIA.

Analytes	C_0 (mg L^{-1})	pseudo-first-order kinetic model		
		q_e (mg g^{-1})	K_2	R^2
ACE	25	24.2	0.047	0.8654
	50	42.7	0.021	0.9012
	100	91.3	0.0052	0.9541
THIA	25	24.9	0.045	0.9021
	50	44.3	0.012	0.9201
	100	92.9	0.0034	0.9257

Table S2. Analysis of adsorption thermodynamics of Fe_3O_4@COF(TBTD-BD)-Au towards ACE and THIA with different initial concentrations.

Analyte	C_0 (mg/L)	ΔG (kJ/mol)			ΔH (kJ/mol)	ΔS (J/mol/K)
		298 K	308 K	318 K		
ACE	15	-5.26	-5.92	-6.43	19.64	81.84
	30	-4.93	-5.53	-6.01		
	50	-4.58	-4.86	-5.42		
	75	-4.01	-4.32	-4.82		
	150	-3.83	-4.15	-4.52		
	200	-2.51	-2.86	-3.43		
THIA	15	-5.06	-6.05	-6.57	17.59	76.26
	30	-4.70	-5.62	-6.33		
	50	-4.41	-4.73	-5.58		
	75	-3.77	-4.29	-4.91		
	150	-3.90	-4.23	-4.49		
	200	-2.71	-2.90	-3.35		
